# Protocol for a cluster randomised controlled trial to evaluate effectiveness of a self-help group intervention to encourage smoke-free homes in slums of Kochi(Kochi Intervention for tobacco smoke free homes-KIFT)

**DOI:** 10.12688/f1000research.141840.2

**Published:** 2024-03-11

**Authors:** Aswathy Sreedevi, Vijayakumar Krishnapillai, Jissa Vinoda Thulaseedharan, Vilma Irazola, Sajitha Krishnan, Akhilesh Kunoor, Jaideep Chanavil Menon, Goodarz Danaei

**Affiliations:** 1 Community Medicine, Amrita Institute of Medical Sciences, Kochi, Kerala, 682041, India; 2 Health Action by People, Thiruvananthapuram, Kerala, 695011, India; 3 Achutha Menon Centre for Health Science Studies, SreeChitra Tirunal Institute of Science and Technology, Thiruvananthapuram, Kerala, 695011, India; 4 Institute for Clinical Effectiveness and Health Policy, Buenos Aires, Argentina, Argentina; 5 Biochemistry, Amrita Institute of Medical Sciences, Kochi, Kerala, 682041, India; 6 Respiratory Medicine, Amrita Institute of Medical Sciences, Kochi, Kerala, 682041, India; 7 Preventive Cardiology, Amrita Institute of Medical Sciences, Kochi, Kerala, 682041, India; 8 Global Health and Population, Harvard T H Chan School of Public Health, Boston, Massachusetts, USA

**Keywords:** Second hand smoke, Intervention, Tobacco smoke-free homes, Self-help group, urinary cotinine, PM 2.5, cRCT, 3 A’s, Fev1/Fev6

## Abstract

**Background:**

Exposure to second hand smoke (SHS) is a cause for heart disease and lung cancer among non- smokers. This cluster randomized control trial will evaluate the effectiveness of a tobacco smoke free home intervention in reducing exposure to second hand smoke.

**Protocol:**

The intervention will be conducted among 30 clusters in urban and peri-urban areas of Kochi, India. The sample size is 300 per arm and 15 clusters to detect a minimal difference of 0.03ng/ml in cotinine levels between groups, at 80% power with a two-sided alfa of 0.05 considering variable cluster size. A baseline survey will be undertaken to identify smokers. Data related to smoking, indoor smoking, nicotine dependence, blood pressure (BP) of smokers, morbidity experienced, and lung volume Fev1/Fev6 of smokers will be measured. Urine cotinine, morbidity, BP of spouse and child will be assessed. Air quality monitors measuring PM2.5 will be placed in homes. Trained self-help group women and frontline health workers will implement the intervention. The intervention will consist of monthly home visits to educate the smoker on the harms of second-hand smoke using 3 A’s. The circle of influencers around the smoking men will also be contacted by the members of self-help group to provide support to stop smoking within homes and to quit. They will then organize two-three meetings of community leaders and heads of women’s groups, present data on harms of SHS, and explain the rationale for establishing smoke free homes in their community for a duration of six months. After the intervention a post assessment will be conducted and this will be repeated after six months.

**Ethics and dissemination:**

The trial protocol was approved by the Institutional Ethical Committee of Amrita Institute of Medical Sciences. Results will be submitted to open access peer reviewed journals and shared with other stakeholders.

**Trial registration:**

CTRI/2021/06/034478

## Introduction

Noncommunicable diseases (NCD) are a major cause of morbidity and mortality in India. The disease transition in India in the past 2 decades is representative of the accelerated epidemiological transition model with a rapid shift to the age of delayed chronic diseases. ^1^ Therefore, in order to halt the epidemic of NCDs, not only should sick persons be treated, but healthy persons also need to be protected. ^2^

In Kerala, 90% of premature mortality in the 15-69 year age group is attributed to NCD. ^3^ Kerala also has the highest rate of coronary artery disease (CAD) ^3^ and diabetes in India, being in the most advanced stage of epidemiologic transition. Over the years the prevalence of Cardiovascular disease has seen an increase with cardiovascular disease accounting for 15.2% of total death in 1990 to 28.1% of total deaths in 2016 and more than half (53.4%) of crude deaths due to CVD were among people younger than 70 yrs. ^4^ While cardiovascular disease risk factors encompass diet, obesity, systolic blood pressure, and air pollution, tobacco use, despite its declining prevalence, consistently ranks among the top six risk factors. ^4^^,^^5^ Another risk factor study in Kerala pointed to the dismal fact that only a fifth of the study population; 20.4% of men and 16.5% were free of any of the seven NCD risk factors studied- tobacco use, alcohol use, obesity, abdominal obesity, raised BP, raised blood glucose or physical inactivity. Three or more risk factors were present in 20.9% and four or more NCD risk factors were present in 6.4% of the participants. ^6^

Globally, tobacco smoking including second-hand smoke (SHS), is one of the three leading risk factors for global disease burden, others being high blood pressure, and household air pollution from solid fuels. ^7^ Though there is a decline in the proportion of people exposed to domestic SHS in India, according to the recent Global Adult Tobacco Survey-2(GATS-2) report, it continues to be high at 38.2% among adults and 39.3% among women. ^8^ Being female, illiterate, poor, homemaker, resident of the rural areas was associated with the highest exposure to SHS at home in GATS-2. ^9^

Chronic exposure to SHS is suggested to be, on average, 80%–90% as harmful as chronic active smoking with a significant dose-response relationship. ^10^^–^^12^ SHS affects the heart and blood vessels, increasing the risk of myocardial infarction, stroke, and emotional changes like depression in non-smokers. Smokers’ children suffer reduced lung function, which continues to affect them in the form of chronic respiratory disorders in adulthood. School-aged children exposed to the harmful effects of second-hand smoke are also at risk for asthma through inflammation of the airways to the lungs. ^13^

Smoking regulations in public places such as hotels, theatres are prohibited and are governed by Section 4 of the Cigarette and other tobacco products Act (COTPA), 2003. ^14^ However, this alone has not resulted in substantial reductions in SHS exposure among women and children. The biggest source of involuntary exposure to tobacco smoke, particularly for children, is smoking by parents and other members of the household. ^15^ Smoking among children in the age group of 12-19 years was found to be higher among urban children and among those from poorer households. ^16^ There are also studies which point to the fact that those who report ever-use of tobacco are more likely to have experienced exposure to SHS at home. ^17^ Studies in urban areas of Kochi showed that SHS exposure from family members among higher secondary school students was 23.2%. ^18^ These are largely from men in the households as women smokers are very low (0.2%-2.2%) in Kerala. ^19^^,^^20^

There are about 300 slums and colonies in Kochi corporation and cardiovascular health issues are the third most common cause of morbidity according to the district government statistics. ^21^The formal health sector encounters slum residents only when they develop late-stage complications of preventable chronic diseases ^22^ due to competing priorities. Slums are a manifestation of the twin challenges of rapid urbanization and the urbanization of poverty. ^23^Despite a National Programme for control of Non-Communicable Diseases (NP-NCD) and laws like the Cigarette and Other Tobacco Products Act (COTPA), NCDs are increasing. Health outcomes are worse in slums and other impoverished settlements. ^23^^,^^24^ Therefore, in states like Kerala which has the highest burden of NCDs, progress in the control of tobacco control needs sustainable solutions embedded in the community. Due to high occupancy rates in all-purpose rooms in slums, smoking within homes can result in a high level of SHS exposure. Hence, the high population density is not only a risk factor but also an opportunity to efficiently reach a vulnerable proportion of the population. ^24^

Community embedded networks such as the “Kudumbashree” (which means welfare of the family) is a community network of women in the State of Kerala working in close coordination with the local self- government. ^25^ Throughout the world, diverse community leaders and organizations has been explored, ^26^^–^^28^ which were feasible with varying degrees of effectiveness. Moreover, a study on a community-based intervention by women’s self-help group members showed a significant reduction in blood pressure levels among people living in urban slums of Kerala, India. ^29^ Also, our initial experience from a smoke free initiative as part of ‘Quit Tobacco international activities’ suggests that “tobacco smoke free homes initiative” were feasible and a six week campaign with self-help group members brought a 30% decline in second hand smoking in a vulnerable fisherman community. ^30^ Therefore, intuitively, the potential impact of such interventions on reduction in second hand exposure and improvement of cardiovascular and respiratory health is large. Hence this study plans to examine the effectiveness of an intervention partnering the self-help groups in reducing the second hand smoke exposure within homes.

Outcomes of the intervention need to measure exposure to second hand smoke. Biomarkers and indicators of environmental pollution were considered. Urinary cotinine level is a widely used biomarker and has a good correlation with daily tobacco consumption and second hand exposure. ^31^ Urine cotinine concentrations are more sensitive than in blood or saliva, making urine a sensitive matrix to detect low-concentration exposure. ^32^ Particulate matter (PM), despite a background concentration of respirable particulate matter (PM) due to cooking and infiltration from outdoors, is considered a good indicator of SHS, at relatively high levels. ^28^^,^^33^ PM < 10 μm enter the respiratory tract, but may be the result of mechanical processes such as construction activities, road dust resuspension and wind whereas PM < 2.5 μm (fine particles) originate primarily from combustion sources such as smoking. ^34^ Quit rates and harm reduction are also outcomes which will be reported as has been observed in other studies. ^35^^,^^36^

In order to assess the impact of the intervention on smokers perse FEV1/FEV6 was measured. This measure is a substitute for FEV1/FVC in diagnosing airflow obstruction and may better predict COPD-related pathology and morbidity. ^37^ The use of 6-s expiratory maneuvers makes handheld spirometry faster and easier, provides a more explicit end-of-test definition. Its use in primary care has also been demonstrated for the detection of COPD in adults. ^38^

Our intention is to enhance the application of evidence in the naturally occurring clusters which are the slums. Each of the slums is a unit with similar sociodemographic features where interventions can be more effective, compliance will be better and contamination less likely. We would like to capture the population level effects of the intervention. Thus, a cluster randomisation trial was chosen.

This study aims to assess the effectiveness of a “smoke-free home” intervention in partnership with women’s self-help groups in the slums of Kochi corporation, Kerala. This is a hybrid type 2 effectiveness-implementation research ^39^ combining the evaluation of effectiveness of the intervention and also assessing the implementation process. We hypothesize that a tobacco smoke-free home initiative in partnership with self-help group women will be effective in significantly reducing SHS in homes and SHS exposure among women and children of those homes over a period of 1 year. The specific aims are:

• To determine the effectiveness of an intervention led by women’s self-help groups to reduce indoor tobacco smoking by measuring urinary cotinine levels and reduction in indoor smoking by measuring PM 2.5 as an indicator for tobacco smoke in the home environment.
• To correlate the urinary cotinine levels with the PM 2.5 in the home environment.
• To measure the effect of the intervention on the FeV1/FeV6 among smokers.
• To explore the impact of the intervention on quitting rate, smoking intensity through in depth interviews and focus group discussions.

## Methods

### Sample selection

We have used the SPIRIT protocol according to guidelines. This will be a cluster-randomized community-based trial. Clusters will be slums with a population of more than 400 or 100 households in the Kochi Corporation and adjacent peri-urban areas. 30 clusters will be selected randomly from the sampling frame of 74 slums and colonies with a population of more than 400 population from a total list of 300 slums and colonies of Kochi Municipal Corporation and adjacent areas. These will be randomly assigned by a computer-generated table to the intervention or control arm with a 1:1 allocation ratio.

### Participants

*Eligibility*: All individuals who have been living in the area for more than 6 months are eligible to participate in the study. In the households the individuals will be screened to identify homes where the male members are between 18-80 years and smoke within the homes. Such male members will be considered for the study and a participation information sheet will be given following which informed written consent will be obtained. In order to determine the impact of passive smoking their spouse or a close female relative will be administered a questionnaire following informed consent. Similarly, children in the house less than 18 years will also be administered the child questionnaire and enrolled in the study after due consent from the legally authorised representative. If there are two children in the study the youngest child older than one years will be enrolled in the study.

*Data collection*: Four semi-structured questionnaires have been developed and pretested. They include household, smoker, spouse and child questionnaires. The household questionnaire is for the household and comprises sociodemographic variables, number of family members, identifying the smokers, number of rooms, ventilation, distance from the road, type of road, any other source of air pollution within 500 m, organic and inorganic waste disposal, cooking fuel used, ventilation in the kitchen, use of mosquito coils, incense sticks, and the presence of pet animals. The smokers’ questionnaire will determine smoking habits, age of initiation of smoking, types of tobacco product used, money spent on tobacco per week, quit attempts, frequency and place of smoking within home, smoking when children are around, nicotine dependence score, awareness of harmful effects of second hand smoke, and who influences smokers the most. Nicotine dependence has been assessed by the Fagenstrom scale. The spouse questionnaire will consist of exposure to second hand smoke at home, practice of indoor smoking, ventilation in the kitchen, exposure to second hand smoke outside the home, cleaning habits, cooking practices, and medication use. The child questionnaire will consist of domains on reports of second-hand exposure at home and outside, history of respiratory morbidity in the past 6 months and history of hospital admission. For older children above 12 years the domains will also consist of history of tobacco use, age of initiation, and awareness regarding harm of second hand smoke. Standard methods of measurement of weight and height will be taken of the smoker, spouse and child. Blood pressure of the smoker and spouse will be measured by an electronic sphygmomanometer in a seated position after ensuring that the person is relaxed. If the reading is ≥140/90 mmHg the measurement will be repeated after 30 min and the average of the two will be taken. In order to measure FeV1/FeV6 the smokers will be instructed to take a deep breath and blow hard and long (6 seconds) till a beep is heard. The mouth should be sealed around the disposable mouthpiece. The measurement will be noted down by the health worker. We will also measure SHS through the air pollution monitor within the home which will measure particulate matter of size 2.5 microns and is a marker for the presence of SHS in the home. The device will be charged and left in the home for recording for 6-8 hours at an elevated place. The machine will record PM 2.5, 10 PM. The sensor will record the readings every 5 minutes and the output will be in the form of a graph. After downloading in the evening it will be given in the next house. This will be done at 6, 10, and 16 months respectively ( Table 1).

**Table 1.** Data collection and outcome measures.

KIFT intervention (2)		Baseline (to)	Post- intervention (1)	Post-Intervention (2)
Demographic info		✓		
Tobacco use within homes		✓	✓	✓
**Smoker**				
Smoking within homes (self-reports)		✓	✓	✓
Fagenstrom nicotine dependence score		✓	✓	✓
No of cigarettes smoked daily		✓	✓	✓
No of persons who have quit			✓	✓
Blood Pressure		✓	✓	✓
BMI		✓	✓	✓
Fev1/Fev6		✓	✓	✓
*Air pollution	PM 2.5	✓	✓	✓
	PM 10	✓	✓	✓
	CO _2_	✓	✓	✓
Women	Cotinine	✓	✓	✓
Child	Cotinine	✓	✓	✓
BMI	Child	✓	✓	✓
Resp. Infection	Child	✓	✓	✓
	Woman	✓	✓	✓
Process evaluation	In depth interview			✓
	FGD			✓

A door-to-door initial survey will be conducted following a training to the data collectors. After ascertaining the households where the male member smokes the interviews of the smoker, wife/close female relative, and child will be conducted. The interviews will be conducted separately for the husband and wife/close relative who is a woman. After the interview, Fev1/FeV6 ratio among the smokers will be assessed with the Vitalograph COPD-6 screener device. The women and child of the household will be asked to keep the urine sample in a sample bottle provided which is then placed in an auto seal pouch. These samples will be collected from all the houses and transported to the laboratory by maintaining cold chain at a temperature between 2-8 degrees C. The urinary cotinine levels among the women and children of the smoking households will be assessed in this urine sample. The cotinine levels will be assessed among the spouse of the smoker or among a woman who stays at home for a longer duration and a child up to 18 years of age. If there is more than one child in the house, the youngest child older than 1 year will be chosen. All houses may not have children; therefore, it is necessary to take both women and children’s cotinine levels. This would also be an important advocacy tool as the children’s cotinine levels can be used to influence the smoker. The urine cotinine level will be measured at 6, 10, and 16 months with the help of Cal biotech’s ELISA kit which will measure cotinine levels by ELISA (solid phase competitive ELISA). The test was calibrated and standardised in a pilot study among households of smokers and non-smokers and differences ascertained.

Quality of data collected will be ensured by training and retraining of the data collectors. The data collection forms are provided. ^40^^,^^41^ The laboratory tests will be carried out after validation. Data entry will be done with back up and range check-ups will be done for data values.

Frequent meetings of the data monitoring committee (DMC) will be carried out to minimise dropout. The members of local self-government will also be taken into confidence to minimise dropout. The data monitoring committee is independent of the sponsors and is situated locally and consists of external senior public health personnel, investigators, sociologists and data analysts. The data regarding unintended effects will also be collected.

### Training

**Baseline survey**: The initial training will be conducted for health workers to carry out the baseline survey. The content of the training will include rapport building, confidentiality, familiarisation with the questionnaires in both English and Malayalam (regional language) for the household, smoker, smoker’s spouse, and child, hands on training on data collection through open data kit (ODK) on a smart phone or tablet. Each day it will be downloaded in Excel and mailed/WhatsApp’d to the program coordinator. Hands on training of the COPD 6 device which measures FeV1/V6 will be given. Monitoring of indoor air pollution for particulate matter will also be explained in a two-day training programme.

### Intervention

**Intervention training of Self Help Group - Accredited Social Health Activist (SHG-ASHA):** A three-day training and sensitisation of self-help group members and ASHAs including outlining expectations in the intervention arm will be conducted. The self-help group are an arm of the Local self-government created for social empowerment, economic empowerment and women empowerment. The list is available with the local self-government and the community is also aware about its members. Similarly the ASHA is the frontline health worker and is known to all the people. Aspects of rapport building, confidentiality, tobacco and types of tobacco products, harms due to second hand smoke, how to change smoking behaviour (3 A’s), benefits of quitting, role and responsibility of ASHA and SHG, and sensitisation regarding the prevailing laws on tobacco and indoor air pollution will be discussed. They will be given a training booklet.

The cluster level consent has been obtained from the local self -government and individual consent will be obtained. Consent will be obtained during baseline survey and also before intervention at the local self-government level.

***Behavioural intervention***

**Figure 1.**
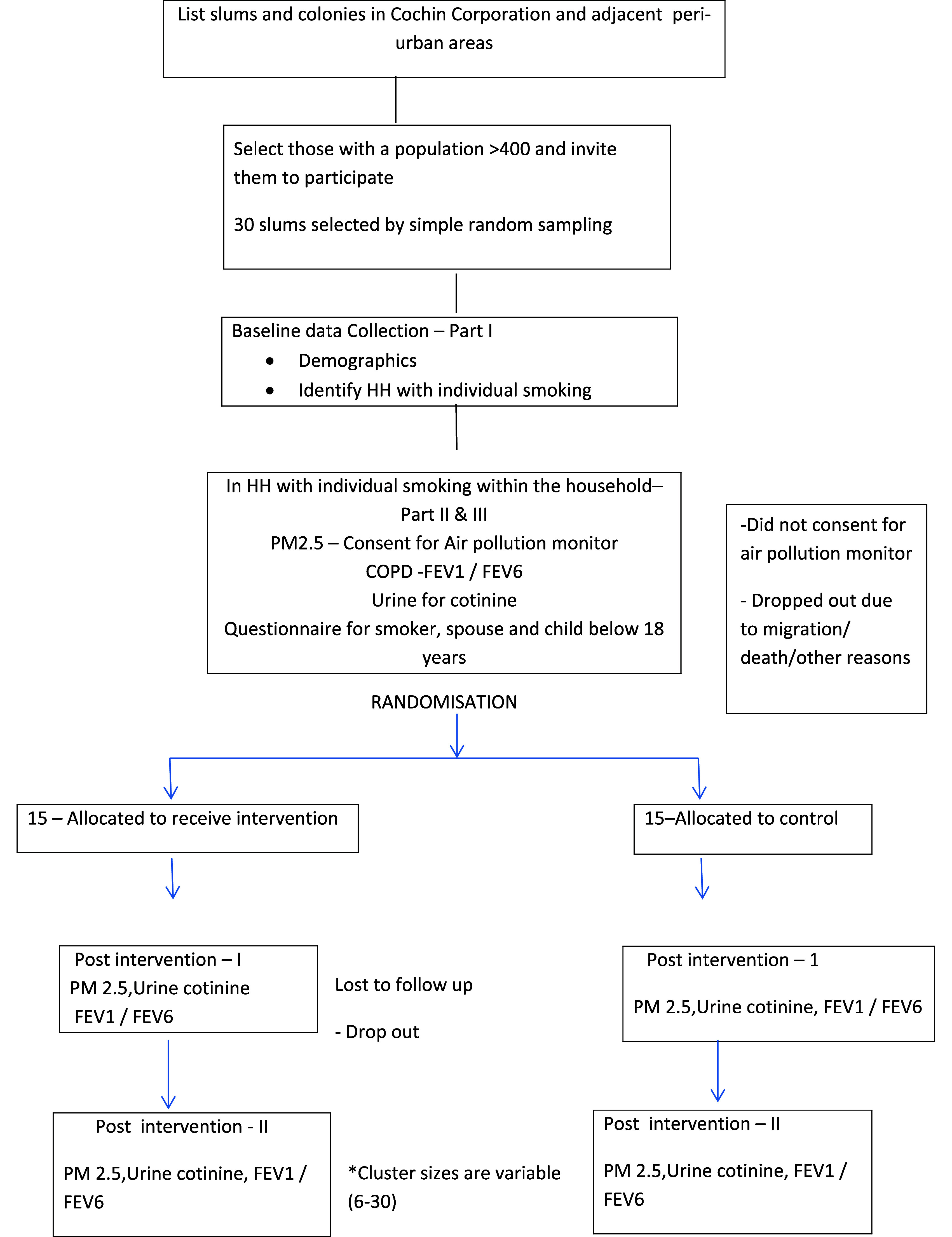
Flow diagram for recruitment and follow up.

*Conceptual framework*: The theoretical basis for the intervention is the socioecological model. The Social Ecological Model (SEM) is a framework for understanding the multifaceted and interactive effects of personal and environmental factors that determine behaviours, and for identifying behavioural and organizational leverage points and intermediaries for health promotion within organizations. ^42^ There are five nested, hierarchical levels of the SEM: individual, interpersonal, community, organizational, and policy/enabling environment ( Figure 1). The most effective approach to public health prevention and control uses a combination of interventions at all levels of the model. At the participant/individual level the self-help group women will visit homes, use the 3 A’s of Ask, Advise and Act to speak to the smokers and their spouses to bring about behaviour change. They will use a flip chart to educate the smoker and his family on the health effects of indoor smoking. At the participant level, which is the interpersonal level in SEM, the self-help group members will talk to the influencers of the smokers. Accredited Social Health Activists (ASHAs) who are the frontline health workers will support the SHGs in implementation of the intervention. At the community and organisational level influence will be brought about by educational meetings in the ward organised by the self-help group member and by the active involvement of the elected representatives of the local self-government, other formal and informal leaders. Testimonials will be shared, the elected member will endorse the idea of smoke free homes, and video clips by leading personalities from the film industry and medical professionals will be shown. At the cluster level, children in the area will also be provided awareness so that they can convey the message in a child to parent approach among the indoor smoker households. Thus it is a composite multipronged intervention delivered by the women self-help group members comprising of face to face educational sessions singly and in groups, using influencers of the tobacco smoker, and educating the children of smokers. The self-help group member and the frontline health worker (ASHA) will be pivotal in the delivery of the intervention.

The training booklet (6-8 pages) has been developed for the ready reference of the SHG women who will deliver the intervention. The training booklet has been prepared by a team comprising a sociologist, health educator, communication specialist, community medicine expert, ASHA, and two SHG workers. The training booklet will be in an easy to understand form. The training booklet will consist of a review of the burden of the problem of tobacco, particularly smoking, the problem of second hand smoke, impact on women and children, and the use of the 3 A’s in the local language. They will also contain advice on the importance of not smoking within the home. The SHG/kudumbasree members will be partners in the development of communication material and will assist in the development of a poster, leaflet, and flip chart in order to ensure clarity, relevance, easy readability and understanding.

In the intervention clusters, an air quality monitoring committee will be formed to support the intervention. This will comprise of the SHG, ASHA, junior public health nurse (female health worker), ward councillor, and members of our team. This committee will guide the intervention. In the initial two months of the intervention the SHG-ASHA team will conduct monthly home visits for educating the smoker, spouse and others in the family on the harms of second-hand smoke using a flip chart. The circle of influencers around the smoking men will also be contacted by the members of the self-help group to provide support to quit. They will then organize a meeting of community leaders and heads of women’s groups, present data on harms of SHS, explain the rationale for establishing smoke free homes in their community, and ask for their assistance in arranging other educational meetings for community members. Two-three such educational meetings will be conducted per cluster where all the household members, particularly that of the smoking households, are encouraged to attend. These meetings will be conducted at different places in the community and doctors from the nearby primary health centres will come and talk about the harms of SHS and importance of becoming a smoke free home community. In these meetings other stakeholders, formal and informal leaders of the community, and local self- government representatives will be invited.

In the next two months home visits will be conducted to paste the smoke free home stickers or distribute the calendars and reinforce the message of not smoking within homes and to get widespread recognition of the movement. Based on the findings of the baseline survey customized messages to quit will be delivered. The SHG-ASHA combined will also communicate the results of the cotinine levels and 2.5pm to the household members. The cotinine levels among the children and women will be an especially powerful tool to motivate and influence men to quit smoking within the homes and then to quit ultimately. If they express an interest to quit, they will be referred to the nearest urban primary health center for tobacco cessation. The influencers will be contacted again to encourage the smoker to quit smoking within home and to quit. Creating a movement and making “not smoking within the house an acceptable sociocultural norm” is the objective. At the end of the intervention as part of the dissemination activity smoke free homes declaration meeting for all community members in the presence of political leaders will be conducted.

*Control arm*: The other arm will get the standard of care which would include the routine programs which are being run by the government as part of the national tobacco control program such as training of health and social workers, NGOs, school teachers, and enforcement officers; Information, Education, and Communication (IEC) activities; school programmes; monitoring of tobacco control laws; coordination with Panchayath Raj Institutions for village level activities; and setting-up and strengthening of cessation facilities including provision of pharmacological treatment facilities at district level. Periodic assessments will be done at 10 and 16 months.

Health workers will conduct end-line surveys soon after the intervention and six months after the intervention in the intervention and control arm with the same questionnaire, urinary cotinine measurement and SHS measurement with an environmental sensor. Thus the measurements will be at 6, 10 months and 16 months respectively.

### Sample size calculation

Based on previous studies we assume an ICC of 0.06. ^43^ To detect a minimal difference of 0.03ng/ml in cotinine levels between groups, ^44^ at 80% power with a two-sided alfa of 0.05, the estimated sample size was 113 indoor smokers per arm for individual randomization. Since one of the studies in the slum area of Ernakulam had found a prevalence of indoor smoking of 16% and considering slums above 100 households it is assumed that the expected number of smokers within household will be 20 per cluster with variability in clusters. Hence we estimated the sample size for unequal cluster size, ^45^ with a CV of 0.45 and VIF=2.4. The estimated sample size was 271 and 14 clusters per arm. Further we assumed a drop-out rate of 10%, the final sample size was rounded to 300 per arm and 15 clusters.

***Randomisation***

The trial has been registered in Clinical Trials Registry – India CTRI/2021/06/034478.

A simple randomisation will be undertaken of the selected slums in the urban and peri-urban areas to receive intervention or not by a computer-generated random number list.

Allocation concealment: Allocation will be based on clusters and the allocation will be done after the baseline survey assessment by a statistician not directly related to the study. There is no allocation concealment. All the indoor smokers will be recruited and they will be selected before randomising clusters.

*Implementation*: Meetings will be conducted with the Kochi Corporation and District Medical Office regarding the objectives of the study. After the baseline survey, which will help to identify the indoor smokers, smoking practices, ventilation issues, and waste management practices, a simple randomisation will be undertaken of the selected slums in the urban and peri-urban areas to receive intervention or not.

The allocation sequence generation and the assignment of participants to the intervention will be carried out by the statistician and the investigators will enrol the participants.

*Blinding*: Blinding may not be possible as a lot of people are involved, local self-government officials, the investigators team, health centres, health workers, frontline workers such as the accredited social health activist, and anganwadi workers. The group of people who collect the outcome data will be blinded.

### Outcomes

***Primary and secondary***

The primary outcome is SHS exposure at homes at 10 months and 16 months as assessed by the change in mean urine cotinine levels among women and or children of the smoker at the individual level. The secondary outcomes will include individual level self-reports of smoking within homes, particulate matter 2.5, harm reduction (reduction in the number of cigarettes smoked), quitting of smoking, blood pressure, Fagenstrom Nicotine dependence score, and comorbidities. Individual level assessment of Fev1/Fev6 will also be carried out with handheld spirometry considering a cut off of 73% to be indicative of COPD which is also a secondary outcome. Forced expiratory volume in 1 second (FEV1) is the maximum amount of air that the subject can forcibly expel during the first second following maximal inhalation. ^46^ Similarly, forced expiratory volume in 6 seconds (FEV6) is the volume of forcibly exhaled air measured during 6 seconds ^47^ which can be measured by handheld spirometry.

Cluster level assessment of 2.5 particulate matter in homes will also be done with air quality monitors. Monitoring and assessing intervention in terms of development of a training booklet, educational material, number of meetings organized, proportion of self-help group members in the area participating and inviting local self-government members to participate.

In order to understand the reasons for success and failure, in-depth interviews of men who have successfully quit smoking within homes, quit smoking completely, ASHAs, and health workers will be conducted. Focus group discussions of wives of smokers who quit smoking within homes and those who reduced the number of cigarettes will be conducted.

*Data analysis and statistical plan*: Data will be entered electronically on a secure file storage system and password protected. Data will be anonymised by assigning a unique identification number to each smoker. Outcome data will be reported in accordance with the Standard Protocol Items: Recommendation for Intervention Trials (SPIRIT). All the smokers will be included in the analysis regardless of reduction in smoking within homes.

***Participant flow***

30 clusters will be randomly assigned into two groups and will receive intended intervention and will be analyzed for primary outcome of urinary cotinine. For each group losses and exclusion of clusters and individual participants will be recorded ( Figure 1).

*Baseline data*: The baseline data will be presented for both clusters and individuals as the risk of chance imbalances are greater with clusters.

*Numbers analyzed*: We plan to analyze 30 clusters with at least 300 indoor smokers in each arm. The urine cotinine of the women and children will be obtained from 300 women and or children in each arm. The indoor home values of 2.5 PM will be obtained from 300 households in each arm. Similarly, the lung function values as indicated by FeV1/FeV6 will be assessed among 300 each in the intervention and control group.

*Outcomes and estimation*: The difference in mean cotinine levels among the clusters in the intervention and control group with 95% CI will be estimated. The intracluster correlation coefficient for each outcome will be reported. Adjusted estimates of clustering will also be provided. Cox’s proportional hazards will be calculated for quitting, harm reduction, blood pressure values. This is to determine the effectiveness of an intervention based on partnering with women’s SHG’s to reduce indoor tobacco smoking by measuring urinary cotinine levels.

A repeated-measures ANOVA will be used to compare the mean urinary cotinine concentration between groups, before and after intervention.

Analysis for self-reported quit rates will be assessed by a Cox proportional hazards model. Generalised linear mixed effect models will be used to test between cluster differences from baseline to each follow up period for each outcome indicator.

We will use an intention to treat approach in analysis. A multilevel model will be used for analysis using the generalized linear mixed effect model which will also adjust for individual covariates. Key variables will be measured at baseline and then compared between arms to check balance; and if both arms are unbalanced with respect to any relevant factors, which can happen given that the unit of randomization is the cluster instead of the individual, those factors will be adjusted for in the final analysis so as to address potential confounding. Analysis for SHS exposure rates and quit rates will be assessed by a Cox proportional hazards model.

• To correlate the urinary cotinine levels with the second-hand smoke in the home environment: correlation and linear regression analysis will be carried out by Pearson’s correlation if it follows a normal distribution and Spearman if it is a non-normal distribution.
  The difference in cotinine levels will be analysed by comparing the urine level of cotinine at the end of follow up, adjusted for the baseline value, by using linear mixed model or GEE to allow for cluster effect.
• To measure the effectiveness of the intervention among smokers by measuring their FeV1/FeV6 using COPD6.
  To determine the effectiveness of the intervention in improvement of lung function the ratio of FeV1/FeV6 will be measured pre- and post-intervention and the mean difference between the groups will be assessed by a paired t-test.
***Qualitative data analysis***
  Interview guides will be developed for in-depth interviews, key-informant interviews and focus group discussions by extensive formative research with literature reviews and taking expert opinions. A list of pre-determined open-ended questions will be used in the interview guides. Probing questions will be asked to explore the topic in-depth. An informed verbal consent will be obtained for participating in the interview and for audio recording the session. The interviews will then be conducted in the regional language, Malayalam. The audio recorded data will be then transcribed verbatim and translated to English. The typed transcripts will then be reviewed for accuracy, completion and familiarization and then manually coded to identify the emerging themes and subthemes. Semi-structured interviews and focus groups will be audio-recorded, transcribed verbatim and anonymised before being coded. Conclusions will then be arrived at by data triangulation.
  Data will be analysed inductively using the grounded theory. Appropriate codes and sub-codes will be generated. Data will be analysed thematically to elicit appropriate themes and sub-themes. Interviews in local languages will be transcribed and the analysis will be conducted by researchers fluent in these languages.
  To understand the impact of the intervention among smokers who quit, those who showed reduced smoking, those who did not stop smoking within homes, and households where the smokers stopped smoking within homes, focus group discussions will be conducted with spouses of the smokers. These focus group discussions will enable the participants to share their experience. Focus group discussions will comprise of 6-8 wives of smokers who quit smoking within homes and or those who reduced the number of cigarrettes/bidis. The other focus groups will be with those who have quit or reduced the number of cigarettes smoked and among those who have not, in order to identify barriers to smoking cessation within homes. The focus groups will also seek to identify factors that affected participation in the meetings, motivation due to the educational sessions, and the factors that most helped to quit smoking within homes or cessation or harm reduction.
  The fidelity of the intervention will be assessed by the number of meetings conducted in an area, the number of smoking persons and household members attending, and the number of influencers of smokers contacted. At the end of the study focus group discussions will be conducted among 6-8 quitters, those who have reduced the number of cigarettes/bidis smoked and those who did not make any change at all. Apart from the focus group discussions, in-depth interviews will be conducted with the self-help group volunteers. These interviews will be conducted at the end of the intervention among about self-help group volunteers who were part of the intervention, councillors, urban primary health centre MO, and a junior public health nurse to identify the key elements that might have helped in effective implementation and good outcomes.
*Ethical issues*: Any adverse events will be recorded and reported to the Chair of the Trial Steering Committee and the Chair of the Ethics Committee. Institutional Ethical Committee approval has been obtained vide letter dated 18-10-2022 from Amrita Institute of Medical Sciences (IEC-AIMS-2022-COMM-284). The protocol V2 was submitted and approved by ethical committee vide above. Informed consent and assent will be obtained from the respective participants by the coordinator of the study. The investigators will only have access to the data.
*Dissemination*: A dissemination will be carried out in Kochi with the participation of all stakeholders. The results of the study will be disseminated to all the stakeholders including the community, state and district NCD control officers. Following this the results will also be disseminated through publication.
*Public involvement*: The local self-government has been taken into confidence and they are fully supportive of this venture. Local steering committees have been formed in the intervention clusters to guide and support the intervention.

### Study status

Currently the baseline data collection is ongoing.

## Discussion

Generalizability: To our knowledge, this is the first trial in India that will investigate the effectiveness of a self-help group led intervention in household where there is indoor smoking. The current study builds on a promising pilot trial which gave the proof of concept of this study. ^30^ Public engagement will continue to be central to this study which enhances the acceptability of the intervention and ensures sustainability.

As far as generalizability is concerned it will be generalizable to similar clusters and individuals inhabiting such clusters rather than to metropolitan areas.

The conceptual framework is the socioecological model which is important in understanding and determining behaviour change. As an implementation research, it seeks to assess impact by the RE-AIM framework ( Table 2). There is mounting interest in the use of theories, models and frameworks to gain insights into the mechanisms by which implementation is more likely to succeed. ^48^ Implementation research is intended to improve people’s health through informed policies, strengthened service delivery. ^49^ The implementation outcome variables are acceptability, adoption, appropriateness, feasibility, fidelity, coverage and sustainability. The large number of contextual factors that affect implementation can interact with each other and change over time which points to the fact that implementation occurs as part of complex adaptive systems. ^48^^,^^49^

**Table 2.** The RE-AIM framework as applicable to KIFT.

RE-AIM dimension	Key questions
Reach	The smoker is expected to benefit. How many smokers were approached and how many were exposed to and benefited from intervention
Effectiveness	The key outcome is cotinine levels in the woman and or child living in the same house
Adoption	The program is applied in selected clusters called slums with the help of the self-help group members supported by frontline health worker-ASHA, elected representative, urban primary Health centre
Implementation	Consistency of delivery of intervention assessed by number of meetings conducted, one house to house visit to the smokers’ household, no of smokers household attended, content of message delivered including 3 A’s
Maintenance	In order to determine maintenance there will be an immediate survey after intervention and then another one at the end of 6 months

Though the RE-AIM network was developed to help make research findings more generalisable when developing and testing interventions, it also helps to plan strategies that can reach most people, effectively achieve and maintain positive health outcomes, be adopted in varied settings and be consistently implemented at a reasonable cost and be sustained in different kind of settings. At the end of the intervention, declaration meeting will be held where Mayor and other officials, elected representatives, health officials and press will attend. If the intervention is found to be successful, this will help in creating policies for smoke free homes.

*Ethical considerations*: Institutional Ethical Committee approval has been obtained vide letter dated 18-10-2022 of Amrita Institute of Medical Sciences (IEC-AIMS-2022-COMM-284). Data collection will be done after getting informed consent and or assent. Assent will also be obtained from children above 11 years and parental consent from those below 11 years.

## Ethics approval and consent to participate

Institutional ethical Committee approval has been obtained vide letter dated 18-10-2022 vide Institutional Ethical Committee of Amrita Institute of Medical Sciences (IEC-AIMS-2022-COMM-284). Informed consent will be taken from all participants or parents/legal guardians of minors to participate in the study.

## Data Availability

No underlying data are associated with this article. figshare: Eng Questionnaire. https://doi.org/10.6084/m9.figshare.24468202.v1 ^26^ This project contains the English language version of the questionnaire that will be used in the study. figshare: Questionnaire for Tobacco Smoke-Free Homes. https://doi.org/10.6084/m9.figshare.24314530.v1 ^27^ This project contains the original questionnaire that will be used in the study. Data are available under the terms of the Creative Commons Attribution 4.0 International license (CC-BY 4.0).
