# Supplementary material for: Protocol for a cluster randomised controlled trial to evaluate effectiveness of a self-help group intervention to encourage smoke-free homes in slums of Kochi(Kochi Intervention for tobacco smoke free homes-KIFT)
Source: F1000Res. 2024 Oct 24;12:1474. Originally published 2023 Nov 15. [Version 3] doi: 10.12688/f1000research.141840.3 (PMC11612615; doi:10.12688/f1000research.141840.3)
Supplement: Peer review history

# Reviewer report: Reviewer response for version 3

**DOI:** 10.5256/f1000research.173773.r334613

**Published:** 2024-10-25

**Authors:** Jayasree AK

**Affiliations:** 1 Govt Medical College, Paraiyaram, India

No new comments.

Is the study design appropriate for the research question?

Yes

Is the rationale for, and objectives of, the study clearly described?

Yes

Are sufficient details of the methods provided to allow replication by others?

Yes

Are the datasets clearly presented in a useable and accessible format?

Not applicable

Reviewer Expertise:

sexual health .

I confirm that I have read this submission and believe that I have an appropriate level of expertise to confirm that it is of an acceptable scientific standard.

# Reviewer report: Reviewer response for version 2

**DOI:** 10.5256/f1000research.163361.r277691

**Published:** 2024-06-12

**Authors:** Jayasree AK

**Affiliations:** 1 Govt Medical College, Paraiyaram, India

**Summary**

 This is an interventional study, a cluster randomized controlled trial to evaluate effectiveness of a self help volunteer group intervention to reduce indoor smoking of people, thereby removing second hand smoke in Kochi slums in Kerala. Baseline study will be conducted to assess cotinine level and other parameters. Intervention is done by trained volunteers and final survey is done in the end of the study after 6 months to assess the effectiveness using the same indicators. It is a community embedded study including multi prong approach and various stakeholders. 

 Is the rationale for, and objectives of, the study clearly described?

 The research is well conceived and rationale is brought out in the introduction. Tobacco and second-hand smoke as the risk factor in the context of epidemiological transition in India and Kerala well explained with sufficient references. Focus on second hand smoke and selection of urban slums also rationalized well. Outcome measures mentioned. Intervention by self help groups and other volunteers indicates the community involvement, as a strength in the implementation research. Objectives are clear. I have the following suggestions.

1. It will be good if the number of self-help group members available or selected for each cluster.
2. Outcome indicators can be further specified as for subjects of SHS in the family (like cotinine), smokers (quit rate) and indoor (already specified as home environment in objectives), in the introduction text and objectives.
3. Relevance of the study in terms of its social and economic benefits in addition to health outcome can be mentioned in one line.

Is the study design appropriate for the research question?

 Yes

 Are sufficient details of the methods provided to allow replication by others?

 Yes. It is well explained. Selection of intervention and control groups, randomization, data collection tools, monitoring indicators are explained well. Data management, quality control and analysis are clear.  Social ecological model as theoretical frame, standardization of tools, use of qualitative data collection, details of training and organizing through committees with various stakeholders illustrate community embeddedness for sustainability. These details may help those who replicate it.

 Queries:

1.  If there are households with only male migrant workers, or households with transgender persons, comprised of smokers and nonsmokers, those families will be included or excluded?
2. Training for survey will be given to volunteers working in both arms. What about training on intervention. Only given to those working with intervention clusters?

Are the datasets clearly presented in a useable and accessible format?

 Not applicable

Is the study design appropriate for the research question?

Yes

Is the rationale for, and objectives of, the study clearly described?

Yes

Are sufficient details of the methods provided to allow replication by others?

Yes

Are the datasets clearly presented in a useable and accessible format?

Not applicable

Reviewer Expertise:

sexual health .

I confirm that I have read this submission and believe that I have an appropriate level of expertise to confirm that it is of an acceptable scientific standard.

# Response

**Published:** 2024-10-20

**Authors:** Aswathy Sreedevi

**Affiliations:** Community Medicine, Amrita Institute of Medical Sciences, Kochi, Kerala, India

1.  If there are households with only male migrant workers, or households with transgender persons, comprised of smokers and nonsmokers, those families will be included or excluded?

Thank you for pointing this out. They will be excluded. Have added that

 2. Training for survey will be given to volunteers working in both arms. What about training on intervention. Only given to those working with intervention clusters?

 Yes , the training is for both arms for the baseline survey. It has been added.

 Training on intervention will only be for those working in the intervention clusters. It has been mentioned under intervention that intervention training is for intervention clusters

 Reply to suggestions.

1. It will be good if the number of self-help group members available or selected for each cluster.

Thank You. Added the number 30 under training

1. Outcome indicators can be further specified as for subjects of SHS in the family (like cotinine), smokers (quit rate) and indoor (already specified as home environment in objectives), in the introduction text and objectives.

Thank you. Cotinine has already been mentioned in introduction. Indoor has been added and the relevance of quit rates and harm reduction among the smokers.

1. Relevance of the study in terms of its social and economic benefits in addition to health outcome can be mentioned in one line.

Very true. Thank you for pointing this out. Added

# Reviewer report: Reviewer response for version 1

**DOI:** 10.5256/f1000research.155322.r236524

**Published:** 2024-02-01

**Authors:** Sophie Orton

**Affiliations:** 1 University of Nottingham, Nottingham, England, UK

This is a protocol for a cluster randomised controlled trial to evaluate the effectiveness of s smoke-free homes intervention in Kochi, India. The protocol describes a complex intervention clearly and concisely.

 The rationale for, and objectives of the study are clearly described, although below I give some recommendations to improve the objectives in particular.

 The study design is appropriate for the research question, and there are sufficient details of the methods to allow replication by others.

 The below comments are recommendations to aid clarity.

 Introduction:

 The introduction nicely sets the scene for this research, and helps the reader understand the setting of the intervention. A few additions are recommended:

 Paragraph 3: What are the smoking regulation in public places, and when were they introduced?

 Are there any estimates of female tobacco use in Kerala? (If very low, this will help to justify why only screening male household members for eligibility)

 SHS exposure among children has been associated with a wide range of health conditions. The introduction would benefit from referencing these, as currently it implies that the main negative health outcomes of SHS exposure is heart disease and lung cancer, where as we know children are particularly vulnerable to other health consequences of SHS exposure.

 “Smoking among children in the age group of 12-19 years showed a higher prevalence among urban children and among those from poorer households’ – it is not clear the relevance of this sentence within this paragraph. Are the authors indicating children/young people are more likely to smoke if exposed to secondhand smoke? Please clarify this statement.

 Paragraph beginning “Community embedded networks…” – are there any other examples internationally of interventions to reduce smoking in the home that have informed this work?

 Please could the authors provide a reference for the term ‘hybrid type 2 effectiveness-implementation research’.

 Objective 1: rather than ‘surrogate’, is urinary cotinine and PM 2.5 an ‘indicator’ for tobacco smoke exposure in the home?

 Objectives generally:

 The introduction at present does not clearly lead to the objectives, i.e. urinary cotinine, PM 2.5 and FeV1/FeV6 have not been introduced previously, and so it is not clear to the general reader why these are the outcomes of importance.

 The introduction would benefit from a paragraph introducing urinary cotinine, PM 2.5 and FeV1/FeV6, what relevance they have to secondhand smoke exposure. How are these measured? How are they associated with secondhand smoke exposure? Are they commonly used measures/indicators for secondhand smoke exposure (please reference previous similar studies that have used these).

 In particular, what is the importance of measuring FeV1/FeV6 among smokers?

 Objective “To explore the impact of the intervention on quitting rate, smoking intensity through in depth interviews and focus group discussions” – is this an intervention to reduce smoking in the home, or to reduce smoking rates? Why would the authors expect a change in smoking quit rates? Can the authors give examples in the background where smoking in the home interventions have also led to increased quit rates among smokers? If this is a secondary outcome, suggest having this as another subheading ‘secondary outcome’.

 Methods

 Please could the authors use an appropriate reporting tool? E.g. CONSORT

 Data collection: please reference which nicotine dependence scale you are using in the text.

 Intervention training of self-help group – who are the self help group and how will they be identified/recruited?

 Outcomes, primary and secondary: will there be cut-points for the urinary cotinine levels? Or change in urinary cotinine?

Is the study design appropriate for the research question?

Yes

Is the rationale for, and objectives of, the study clearly described?

Yes

Are sufficient details of the methods provided to allow replication by others?

Yes

Are the datasets clearly presented in a useable and accessible format?

Not applicable

Reviewer Expertise:

Secondhand smoke exposure in the home, smoking in pregnancy and postpartum

I confirm that I have read this submission and believe that I have an appropriate level of expertise to confirm that it is of an acceptable scientific standard, however I have significant reservations, as outlined above.

# Response

**Published:** 2024-03-04

**Authors:** Aswathy Sreedevi

**Affiliations:** Community Medicine, Amrita Institute of Medical Sciences, Kochi, Kerala, India

Paragraph 3: What are the smoking regulation in public places, and when were they introduced? Are there any estimates of female tobacco use in Kerala? (If very low, this will help to justify why only screening male household members for eligibility)

 Smoking regulations in public places such as hotels, theatres are prohibited and are governed by Section 4 of the Cigarette and other tobacco products Act(COTPA),2003. ^14 ^However, this alone has not resulted in substantial reductions in SHS exposure among women and children.

 These are largely from men in the households as women smokers are very  low (0.2%-2.2%) in Kerala. ^19,20^ According to GATS-2( https://ntcp.mohfw.gov.in/assets/document/surveys-reports-publications/GATS-2-FactSheet.pdf )it was 0.2%and according to NFHS survey 5 in 2019-2020 it  was only 2.2%  ( https://www.im4change.org/docs/Kerala%20NFHS-5%20Factsheet.pdf).

 SHS exposure among children has been associated with a wide range of health conditions. The introduction would benefit from referencing these, as currently it implies that the main negative health outcomes of SHS exposure is heart disease and lung cancer, where as we know children are particularly vulnerable to other health consequences of SHS exposure.

 Thank you. The following paragraph has been added. “Chronic exposure to SHS is suggested to be, on average, 80%–90% as harmful as chronic active smoking with a significant dose-response relationship. ^10-12^ SHS affects the heart and blood vessels, increasing the risk of myocardial infarction, stroke, and emotional changes like depression in non-smokers. Smokers’ children suffer reduced lung function, which continues to affect them in the form of chronic respiratory disorders in adulthood. School-aged children exposed to the harmful effects of second-hand smoke are also at risk for asthma through inflammation of the airways to the lungs. ^13^”

  “Smoking among children in the age group of 12-19 years showed a higher prevalence among urban children and among those from poorer households’ – it is not clear the relevance of this sentence within this paragraph. Are the authors indicating children/young people are more likely to smoke if exposed to secondhand smoke? Please clarify this statement.

 Yes-that’s what we are trying to say. Hope the following statement makes it clear.

 “Smoking among children in the age group of 12-19 years was found to be higher  among urban children and among those from poorer households. ^16 ^There are also studies which point to the fact that those who report ever-use of tobacco are more likely to have experienced exposure to SHS at home. ^17”^

 Paragraph beginning “Community embedded networks...” – are there any other examples internationally of interventions to reduce smoking in the home that have informed this work?

 They have been added “Throughout the world, diverse community leaders and organizations has been explored, ^26,27,28^ which were feasible with varying degrees of effectiveness. Moreover, a study on a community-based intervention by women’s self-help group members showed a significant reduction in blood pressure levels among people living in urban slums of Kerala, India. ^29 ^Also, our initial experience from a smoke free initiative as part of ‘Quit Tobacco international activities’ suggests that “tobacco smoke free homes initiative” were feasible and a six week campaign with self-help group members brought a 30% decline in second hand smoking in a vulnerable fisherman community. ^30”^

 Please could the authors provide a reference for the term ‘hybrid type 2 effectiveness- implementation research’.

 Has been provided

 Objective 1: rather than ‘surrogate’, is urinary cotinine and PM 2.5 an ‘indicator’ for tobacco smoke exposure in the home?

 Thank you. The word surrogate has been replaced by indicator

 Objectives generally:

 The introduction at present does not clearly lead to the objectives, i.e. urinary cotinine, PM 2.5 and FeV1/FeV6 have not been introduced previously, and so it is not clear to the general reader why these are the outcomes of importance.

 Thank you for pointing this omission out .Now they have been introduced.

 The introduction would benefit from a paragraph introducing urinary cotinine, PM 2.5 and FeV1/FeV6, what relevance they have to secondhand smoke exposure. How are these measured? How are they associated with secondhand smoke exposure? Are they commonly used measures/indicators for secondhand smoke exposure (please reference previous similar studies that have used these).

 Outcomes of the intervention need to measure exposure to second hand smoke. Biomarkers and indicators of environmental pollution were considered. Urinary cotinine level is a widely used biomarker and has a good correlation with daily tobacco consumption and second hand exposure. ^31^ Urine cotinine concentrations are more sensitive than in blood or saliva, making urine a sensitive matrix to detect low-concentration exposure. ^32,44 ^Particulate matter(PM), despite a background concentration of respirable particulate matter (PM) due to cooking and infiltration from outdoors, is considered a good indicator of  SHS, at relatively high levels. ^28,33^  PM < 10 μm enter the respiratory tract, but may be the result of mechanical processes such as construction activities, road dust resuspension and wind whereas PM < 2.5 μm (fine particles) originate primarily from combustion sources such as smoking. ^34^ Quit rates and harm reduction are also  outcomes which will be reported as has been observed in other studies. ^35,36^ . They have been added with studies that have used them ( ^33,44^)

 In particular, what is the importance of measuring FeV1/FeV6 among smokers?

 In order to assess the impact of the intervention on smokers perse FEV1/FEV6 was measured. This measure is a substitute for FEV1/FVC in diagnosing airflow obstruction and may better predict COPD-related pathology and morbidity. ^37^ The use of 6-s expiratory maneuvers makes handheld spirometry faster and easier, provides a more explicit end-of-test definition. Its use in primary care has also been demonstrated for the detection of COPD in adults. ^38^

 Objective “To explore the impact of the intervention on quitting rate, smoking intensity through in depth interviews and focus group discussions” – is this an intervention to reduce smoking in the home, or to reduce smoking rates? Why would the authors expect a change in smoking quit rates? Can the authors give examples in the background where smoking in the home interventions have also led to increased quit rates among smokers? If this is a secondary outcome, suggest having this as another subheading ‘secondary outcome’.

 It has been mentioned under secondary outcome only.

 “Quit rates and harm reduction are also  outcomes which will be reported as has been observed in other studies. ^35,36^”

 A study which investigated whether cessation rates varied by changes in home smoking ban implementation between enrollment and 7-month follow-up found a higher cessation among those who Among callers who did not live with other smokers in the home, the highest odds ratio of 30-day cessation was observed among  callers. (Yuan NP, Nair US, Crane TE, Krupski L, Collins BN, Bell ML. Impact of changes in home smoking bans on tobacco cessation among quitline callers. Health Educ Res. 2019 Jun 1;34(3):345-355. doi: 10.1093/her/cyz008. PMID: 30932151; PMCID: PMC6510015.)Another study in 20 LMIC reported that Smokefree homes were associated with reduced cigarettes per day in all daily smokers. (Daniel Owusu, Megan Quinn, Kesheng Wang, Faustine Williams, Hadii M. Mamudu, Smokefree home rules and cigarette smoking intensity among smokers in different stages of smoking cessation from 20 low-and-middle income countries,Preventive Medicine, Volume 132,2020,106000,ISSN 0091-7435).

 Methods

 Please could the authors use an appropriate reporting tool? E.g. CONSORT

 Thank you. We have used the SPIRIT guidelines according to the journal guidelines

 Data collection: please reference which nicotine dependence scale you are using in the text.

 Thank you for pointing this out. This has now been added -Fagenstrom dependence scale.

 Intervention training of self-help group – who are the self help group and how will they be identified/recruited?

 The self- help group are an arm of the Local self- government created for social empowerment, economic empowerment and women empowerment. The list is available with the local self- government[DS1]  and the community is also aware about its members. Similarly the ASHA is the frontline health worker and is known to all the people.

 Outcomes, primary and secondary: will there be cut-points for the urinary cotinine levels? Or change in urinary cotinine?

 Change in mean/median values will be considered. It has been added

 Methods

 We have used the SPIRIT protocol as is the guideline of the F1000 journal.

 Data collection: the Fagenstrom nicotine dependence test.

 The self- help group are an arm of the Local self- government created for social empowerment, economic empowerment and women empowerment. The list is available with the local self- government and the community is also aware about its members.

# Reviewer report: Reviewer response for version 1

**DOI:** 10.5256/f1000research.155322.r224000

**Published:** 2024-01-27

**Authors:** Brijesh Sathian Hamad

**Affiliations:** 1 Hamad Medical Corporation, Doha, Qatar

- Provide more literature review for the gap analysis and importance to Kerala. What is the current burden of noncommunicable diseases (NCDs) in India, particularly in Kerala, and how has it evolved over time?
- How does exposure to second-hand smoke (SHS) contribute to the burden of NCDs, especially in vulnerable populations like children, women, and the elderly?
- What are the main risk factors for cardiovascular diseases in Kerala, and how do these risk factors vary among different demographic groups?
- How effective have past tobacco control measures been in reducing SHS exposure, particularly within households, in India, and what are the ongoing challenges? What role do slums play in contributing to the burden of cardiovascular health issues, and how does the formal healthcare sector currently address health concerns in these communities?
- What is the significance of community networks, such as "Kudumbashree," in the context of public health interventions, and how have they been utilized in previous health-related initiatives in Kerala?
- Can community-based interventions, especially those involving women's self-help groups, effectively contribute to reducing SHS exposure in households and improving cardiovascular health outcomes?
- How has the implementation of smoke-free initiatives, particularly those involving self-help group members, demonstrated feasibility and effectiveness in previous studies or initiatives?
- What evidence supports the hypothesis that a "tobacco smoke-free home" initiative in partnership with self-help group women can significantly reduce SHS exposure in homes and improve health outcomes over a one-year period? 
- Provide more details about the sample size calculation and the ICC selection. How will be the inter cluster variation measured? provide more details about the machine which will be used to measure FeV1/FeV6. 

Is the study design appropriate for the research question?

Yes

Is the rationale for, and objectives of, the study clearly described?

Yes

Are sufficient details of the methods provided to allow replication by others?

No

Are the datasets clearly presented in a useable and accessible format?

Not applicable

Reviewer Expertise:

Clinical trials

I confirm that I have read this submission and believe that I have an appropriate level of expertise to confirm that it is of an acceptable scientific standard, however I have significant reservations, as outlined above.

# Response

**Published:** 2024-03-04

**Authors:** Aswathy Sreedevi

**Affiliations:** Community Medicine, Amrita Institute of Medical Sciences, Kochi, Kerala, India

- Provide more literature review for the gap analysis and importance to Kerala. What is the current burden of noncommunicable diseases (NCDs) in India, particularly in Kerala, and how has it evolved over time?

Thank you. Has been provided now in the first two paragraphs of introduction

 “Noncommunicable diseases (NCD) are a major cause of morbidity and mortality in India. The disease transition in India in the past 2 decades is representative of the accelerated epidemiological transition model with a rapid shift to the age of delayed chronic diseases. ^1 ^Therefore, in order to halt the epidemic of NCDs, not only should sick persons be treated, but healthy persons also need to be protected. ^2^

 In Kerala, 90% of premature mortality in the 15-69 year age group is attributed to NCD. ^3 ^Kerala also has the highest rate of coronary artery disease (CAD) ^3^and diabetes in India, being in the most advanced stage of epidemiologic transition. Over the years the prevalence of Cardiovascular disease has seen an increase with cardiovascular disease accounting for 15.2% of total death in 1990 to 28.1% of total deaths in 2016 and more than half (53.4% ) of crude deaths due to CVD were among people younger than 70 yrs. ^4^ While cardiovascular disease risk factors encompass diet, obesity, systolic blood pressure, and air pollution, tobacco use, despite its declining prevalence, consistently ranks among the top six risk factors. ^4,5^ Another risk factor study in Kerala pointed to the dismal fact that only a fifth of the study population; 20.4% of men and 16.5% were free of any of the seven NCD risk factors studied- tobacco use, alcohol use, obesity, abdominal obesity, raised BP, raised blood glucose or physical inactivity. Three or more risk factors were present in 20.9% and four or more NCD risk factors were present in 6.4% of the participants. ^6^”

- How does exposure to second-hand smoke (SHS) contribute to the burden of NCDs, especially in vulnerable populations like children, women, and the elderly?

Chronic exposure to SHS is suggested to be, on average, 80%–90% as harmful as chronic active smoking with a significant dose-response relationship. ^10-12^ SHS affects the heart and blood vessels, increasing the risk of myocardial infarction, stroke, and emotional changes like depression in non-smokers. Smokers’ children suffer reduced lung function, which continues to affect them in the form of chronic respiratory disorders in adulthood. School-aged children exposed to the harmful effects of second-hand smoke are also at risk for asthma through inflammation of the airways to the lungs. ^13^

- What are the main risk factors for cardiovascular diseases in Kerala, and how do these risk factors vary among different demographic groups?

While cardiovascular disease risk factors encompass diet, obesity, systolic blood pressure, and air pollution, tobacco use, despite its declining prevalence, consistently ranks among the top six risk factors. ^4,5^ Another risk factor study in Kerala pointed to the dismal fact that only a fifth of the study population; 20.4% of men and 16.5% were free of any of the seven NCD risk factors studied- tobacco use, alcohol use, obesity, abdominal obesity, raised BP, raised blood glucose or physical inactivity. Three or more risk factors were present in 20.9% and four or more NCD risk factors were present in 6.4% of the participants. ^6^

- How effective have past tobacco control measures been in reducing SHS exposure, particularly within households, in India, and what are the ongoing challenges? What role do slums play in contributing to the burden of cardiovascular health issues, and how does the formal healthcare sector currently address health concerns in these communities?
- Not much .The COTPA Act and its limitation in addressing exposure at smoke and the exposure as reported among school children has been mentioned. The tobacco control laws particularly section 4 apply to public places and as has been mentioned in para2 biggest source is involuntary exposure at home
- What is the significance of community networks, such as "Kudumbashree," in the context of public health interventions, and how have they been utilized in previous health-related initiatives in Kerala?

This has been added.

- Can community-based interventions, especially those involving women's self-help groups, effectively contribute to reducing SHS exposure in households and improving cardiovascular health outcomes?
- Thank you for pointing this out. They have been added and listed.

Throughout the world, diverse community leaders and organizations has been explored, ^26,27,28^ which were feasible with varying degrees of effectiveness. Moreover, a study on a community-based intervention by women’s self-help group members showed a significant reduction in blood pressure levels among people living in urban slums of Kerala, India. ^29 ^Also, our initial experience from a smoke free initiative as part of ‘Quit Tobacco international activities’ suggests that “tobacco smoke free homes initiative” were feasible and a six week campaign with self-help group members brought a 30% decline in second hand smoking in a vulnerable fisherman community. ^30 ^Therefore, intuitively, the potential impact of such interventions on reduction in second hand exposure and improvement of cardiovascular and respiratory health is large. Hence this study plans to examine the effectiveness of an intervention partnering the self-help groups in reducing the second hand smoke exposure within homes.

- How has the implementation of smoke-free initiatives, particularly those involving self-help group members, demonstrated feasibility and effectiveness in previous studies or initiatives?
- Added
- What evidence supports the hypothesis that a "tobacco smoke-free home" initiative in partnership with self-help group women can significantly reduce SHS exposure in homes and improve health outcomes over a one-year period?
- The evidence as below
- Throughout the world, diverse community leaders and organizations has been explored, ^26,27,28^ which were feasible with varying degrees of effectiveness. Moreover, a study on a community-based intervention by women’s self-help group members showed a significant reduction in blood pressure levels among people living in urban slums of Kerala, India. ^29 ^Also, our initial experience from a smoke free initiative as part of ‘Quit Tobacco international activities’ suggests that “tobacco smoke free homes initiative” were feasible and a six week campaign with self-help group members brought a 30% decline in second hand smoking in a vulnerable fisherman community. ^30 ^Therefore, intuitively, the potential impact of such interventions on reduction in second hand exposure and improvement of cardiovascular and respiratory health is large. Hence this study plans to examine the effectiveness of an intervention partnering the self-help groups in reducing the second hand smoke exposure within homes.
- Provide more details about the sample size calculation and the ICC selection. How will be the inter cluster variation measured?
- Thank you for this question. The reference for ICC has been added.
- The mode of calculation of sample size is as below.
- **Ref:** **Hemming et al. BMC Medical Research Methodology 2011, 11:102**
- **Sample size under individual randomization **
- mean 1: 0.09
- mean 2: 0.06
- standard deviation 1: 0.08 
- standard deviation 2: 0.08
- significance level: 0.05
- power: 0.80
- n= 113 per arm (using equation3)
- **Standard sample size under cluster randomization (where the cluster size are unequal)**
- Here, the SD of cluster size is approximately 9 (range/4 = (40-4)/4 =36/4 =9)
- And an average cluster size of 20. Hence,
- Coefficient of variation of cluster sizes (CV) = 9/20 = 0.45
- Variance Inflation Factor (Design effect) = 2.4 (using equation 6)
-  Then the required sample size per arm, n _c_= n xVIF (using equation 8) = 113*2.4 =271.2 rounded to 271
- Number of clusters = 113*2.4/20 (using equation 10) = 13.6 rounded to 14 clusters per arm
- Again adjusting for a 10% drop out rate, the sample size per arm is increased to 300
- provide more details about the machine which will be used to measure FeV1/FeV6.

The portable spirometer is a device manufactured by Vitalograph and used to measure lung capacity. This has been added

 “After the interview, Fev1/FeV6 ratio among the smokers will be assessed with the Vitalograph COPD-6 screener device”

 The Vitalograph copd-6 is an accurate and cost effective ‘case selection’ or screening device that identifies potential cases of COPD

- Identifies those at risk of COPD at the pre-symptomatic stage
- Screens out those whose FEV1 is normal, without the risk of false negatives
- Displays the key parameters for clinical interpretation including obstructive index and COPD classification (stage I – IV)
-  Displays FEV1, FEV6, ratio and % predicted, obstructive index, COPD classification and lung age
- Detachable flowhead makes cleaning simple
- Built-in quality of blow indicator
- Designed for multi-subject use
